# Rifampin monotherapy for children with idiopathic infantile hypercalcemia

**DOI:** 10.1016/j.jsbmb.2023.106301

**Published:** 2023-03-27

**Authors:** Nina Lenherr-Taube, Michelle Furman, Esther Assor, Kenneth Thummel, Michael A. Levine, Etienne Sochett

**Affiliations:** a Department of Pediatrics, Division of Endocrinology, The Hospital for Sick Children, University of Toronto, Toronto, Ontario, Canada; b Department of Pediatrics, Division of Endocrinology, University Children’s Hospital Zurich, Zurich, Switzerland; c Department of Pharmaceutics, University of Washington, Seattle, WA, United States; d Division of Endocrinology and Diabetes, The Children’s Hospital of Philadelphia and Department of Pediatrics, University of Pennsylvania Perelman School of Medicine, Philadelphia, PA, United States

**Keywords:** Idiopathic infantile hypercalcemia, Rifampin, Vitamin D, CYP24A1

## Abstract

Idiopathic Infantile Hypercalcemia (IIH) is characterized by hypercalcemia and hypercalciuria owing to PTH-independent increases in circulating concentrations of 1,25(OH)2D. At least 3 forms of IHH can be distinguished genetically and mechanistically: infantile hypercalcemia-1 (Hypercalcemia, Infantile, 1; HCINF1) due to CYP24A1 mutations results in decreased inactivation of 1,25(OH)2D; HCINF2 due to SLC34A1 mutations results in excessive 1,25(OH)2D production; and HCINF3 in which a variety of gene variants of uncertain significance (VUS) have been identified and where the mechanism for increased 1,25 (OH)2D is unclear. Conventional management with dietary calcium and vitamin D restriction has only limited success. Induction of the P450 enzyme CYP3A4 by rifampin can provide an alternate pathway for inactivation of 1,25(OH)2D that is useful in HCINF1 and may be effective in other forms of IIH.

We sought to assess the efficacy of rifampin to decrease levels of serum 1,25(OH)2D and calcium, and urinary calcium concentrations in subjects with HCINF3, and to compare the response to a control subject with HCINF1.

Four subjects with HCINF3 and the control subject with HCINF1 completed the study using rifampin 5 mg/kg/day and 10 mg/kg/day each for 2 months separated by a 2-month washout period. Patients had age-appropriate intake of dietary calcium plus 200 IU vitamin D/day. Primary outcome was efficacy of rifampin to lower serum concentrations of 1,25(OH)2D. The secondary outcomes included the reduction of serum calcium, urinary calcium excretion (as random urine calcium: creatinine (ca:cr) ratio) and serum 1,25(OH)2D/PTH ratio.

Rifampin was well tolerated and induced CYP3A4 at both doses in all subjects. The control subject with HCINF1 showed significant response to both rifampin doses with decreases in the serum concentration of 1,25 (OH)2D and the 1,25(OH)2D/PTH ratio while the serum and urine ca:cr levels were unchanged. The four patients with HCINF3 showed reductions in 1,25(OH)2D and urinary ca:cr after 10 mg/kg/d, but hypercalcemia did not improve and there were variable responses in 1,25(OH)2D/PTH ratios. These results support further longer-term studies to clarify the usefulness of rifampin as a medical therapy for IIH.

## 1. Introduction

Idiopathic Infantile Hypercalcemia (IIH) is a rare disorder in which PTH-independent elevation of the serum concentration of 1,25(OH)2D results in hypercalcemia and/or hypercalciuria [1–3]. The clinical presentation is variable [4,5]; the most severe form is characterized by symptomatic hypercalcemia, failure to thrive, and nephrocalcinosis [1, 2,6]. Less severe forms can present with more subtle symptoms such as feeding difficulties, constipation, and poor weight gain [7]. Two forms of IIH have been distinguished based on genetics and mechanisms: in Hypercalcemia, Infantile 1 (HCINF1; OMIM 143880) biallelic loss-of-function mutations in *CYP24A1* impair inactivation of 1,25(OH) 2D; in HCINF2 (OMIM 616963) biallelic loss-of-function mutations in *SLC34A1* lead to excessive synthesis of 1,25(OH)2D. It is likely that additional forms of IIH exist, particularly as some IIH patients lack pathogenic *CYP24A1* or *SLC34A1* variants but carry one of more variants of unknown significance (VUS) in genes that have been associated with elevated serum levels of 1,25(OH)2D and/or hypercalciuria. We have proposed calling this group of disorders “HCINF3”, but admittedly the underlying pathophysiological mechanism(s) for these conditions remains uncertain [3, 8–11].

**Fig. 1.**
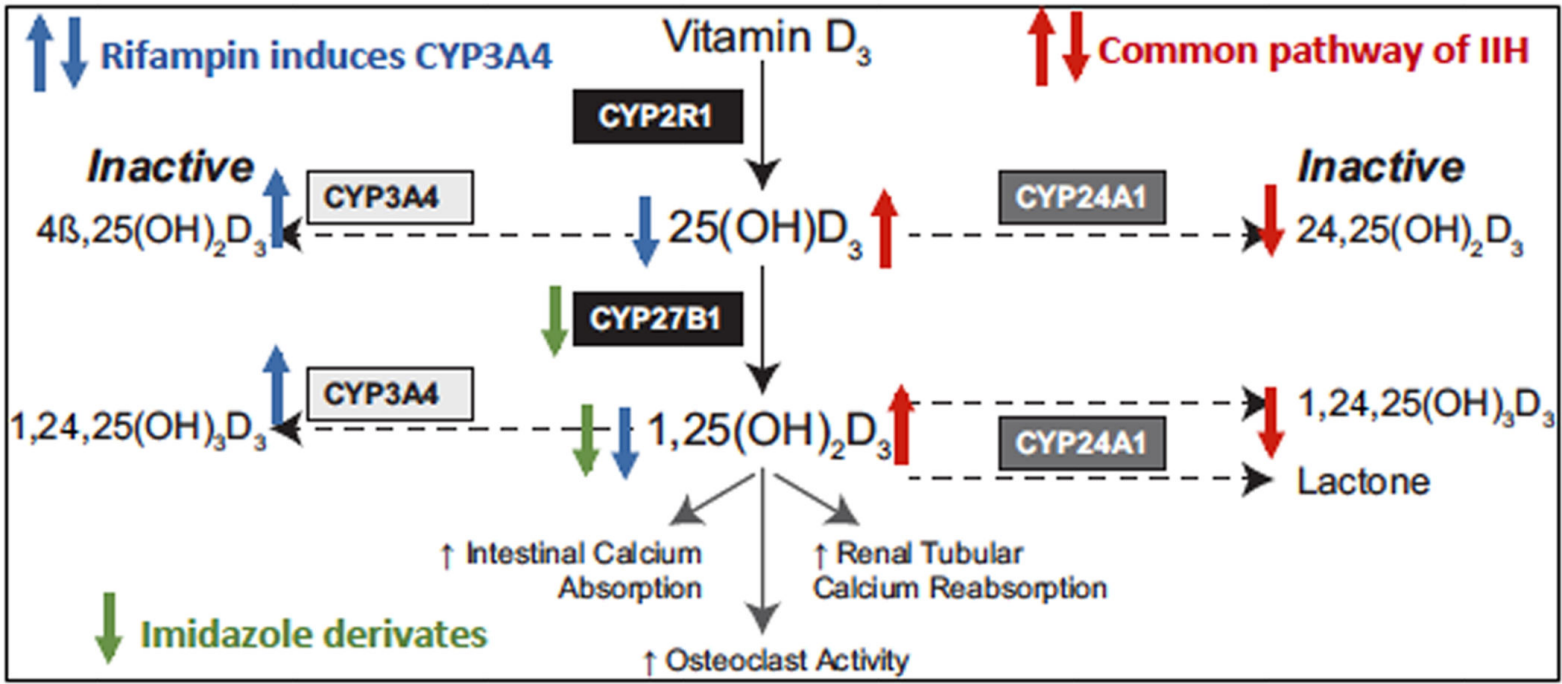
Vitamin D metabolism.

Patients with IIH have life-long risks for renal calcification and renal damage. Management of IIH is based on chronic restriction of dietary calcium and vitamin D. With this approach, hypercalcemia and hypercalciuria improves in some, but in many patients, elevated serum levels of 1,25(OH)2D, and the 1,25(OH)2D to PTH (1,25(OH)2D/PTH) ratio, (an index for PTH-independent accumulation of 1,25(OH)2D) providing evidence that the underlying pathophysiology remains active [7]. In HCINF1, the azoles ketoconazole and fluconazole have been used to inhibit CYP27B1 activity and thereby reduce the conversion of 25(OH)D to 1,25(OH)2D [12,13]. Some efficacy has been shown, however, long-term usage is constrained because of concerns of liver damage and inhibition of other P450 enzymes with the development of low serum levels of serum cortisol and/or testosterone. A second approach has been to use the antibiotic rifampin to induce overexpression of CYP3A4 [14], a P450 microsomal enzyme that is predominantly expressed in the liver and intestine and known to be important in the metabolism of many exogenous drugs, xenobiotics, and steroid hormones [15] (Fig. 1). A recurrent gain-of-function variant in *CYP3A4* has been associated with vitamin D rickets [15], and induction of CYP3A4 by rifampin and other medications can similarly lead to vitamin D deficiency as a side effect [14,16]. These observations have suggested that targeted overexpression of CYP3A4 can provide an alternate pathway for inactivation of vitamin D metabolites, including 1,25 (OH)2D. In a proof of principle pilot study, Hawkes et al. showed that rifampin treatment could improve the biochemical and clinical outcomes in two young patients with HCINF1 due to biallelic *CYP24A1* mutations [17]. More recently rifampin was shown to be a safe and effective long-term treatment for HCINF1 that induced normalization of serum calcium and improved renal function in a single adult patient [18].

By contrast, patients with HCINF2 experienced renal phosphate-wasting that leads to suppression of FGF23 levels with consequent generation of 1,25(OH)2D. This pathophysiology has led some investigators to suggest phosphate supplementation, particularly for those patients who have serum phosphate levels that are low or borderline low [9]. However, the safety and efficacy of long-term phosphate supplementation in these patients remains unclear. Currently, there are no specific pharmacological recommendations for patients with other forms of IIH. Therefore, the aim of this study was to determine the efficacy, safety, and impact on vitamin D metabolism of short-term rifampin treatment in children with mild hypercalcemia due to HCINF3 and compare this with the response in a control patient with HCINF1.

## 2. Material and methods

### 2.1. Study design

This was an open-label, single-arm, pilot phase II study evaluating the efficacy and safety of rifampin in patients with mild HCINF3 due to abnormal vitamin D metabolism; it was conducted from January 2018 to August 2021. Given the limited available patient pool, randomization of participants was not feasible. To satisfy the phase II nature of this study, dose escalation was necessary allowing for adjustments to be made based on the observed effect of the starting rifampin dose. The study was approved by institutional review boards (NCT:03384121), with informed consent and assent procedures followed in accordance with the Declaration of Helsinki.

### 2.2. Enrollment criteria

Inclusion criteria included the following: patients between 6 months-17 years of age followed in the Calcium Clinic at the Hospital for Sick Children with 12–16 months of documented increased serum levels of calcium (total and/or ionized) above upper limit of the reference range for age, elevated (or high normal) serum concentration of 1,25 (OH)2D, and low serum PTH level, with or without a genetic variant associated with IIH. Participants were excluded if there was a history of allergy to rifampin, significant co-morbidities, tuberculosis, pregnancy, variant in CYP24A1 or SLC34A1, and/or if they were taking any medications/supplements known to interact with CYP3A4, or 1,25(OH)2D. One subject with HCINF1 who carried biallelic pathogenic variants in *CYP24A1* was enrolled as a control.

### 2.3. Study treatment

**Fig. 2.**
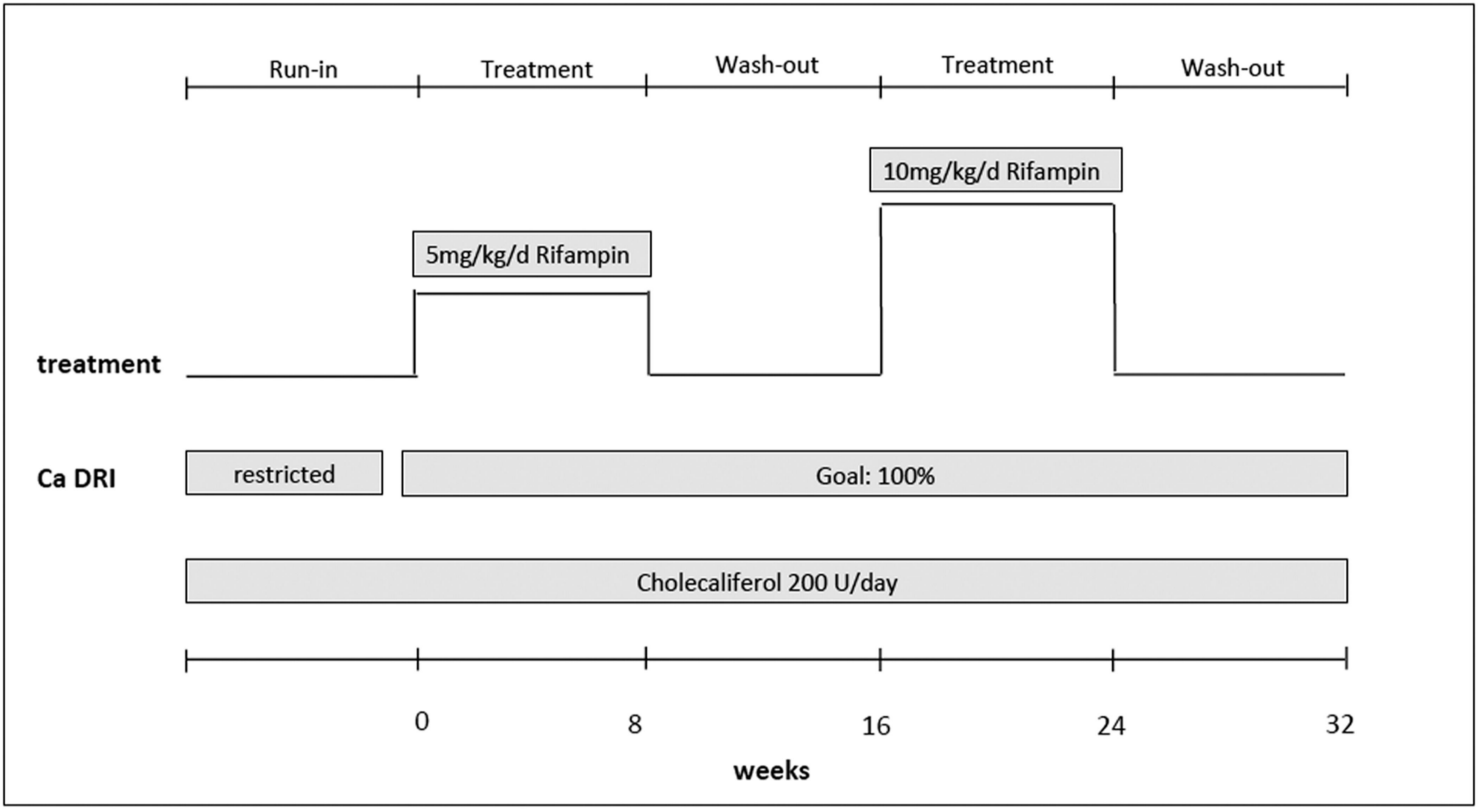
Study design.

At initiation of study, rifampin was started at 5 mg/kg/day orally for two months (up to a maximum of 300 mg). This was followed by a washout period of two months. Rifampin was then restarted at 10 mg/kg/day (up to a maximum of 600 mg) for another two months. This was then followed by a second washout period of two months, with participants returning to site for the end of study visit (Fig. 2). In addition to the daily rifampin, participants were instructed to take a daily vitamin D supplement of 200 IU, to avoid vitamin D-fortified foods, sunlight exposure and to consume an age-appropriate daily recommended intake (DRI) for calcium.

Duration of treatment was based on [1] limited clinical reports documented in the literature [17,18] [2] pharmacodynamics of rifampin showing that complete induction of drug-metabolizing enzymes is reached in about one week with resolution of induction in approximately 2 weeks [17,18], and [3] goal of limiting the possibility that extraneous factors such as change in season and/or intercurrent illness might confound the response. The 2-month washout period was determined based on pharmacodynamics of rifampin [17–19]. In children, peak serum levels range from 3.5 to 15 mcg/ml, with a study showing the half-life of rifampin was 1–3.8 h after 600 mg oral dose [19,20].

The optimal dose for treating IIH is not known. Standard dosing for rifampin in pediatrics varies between 10 and 20 mg/kg/day. Prior experience using 10 mg/kg/ day [17] was used as the guide for likely effective treatment, and a lower dose of 5 mg/kg was also used to better understand the lowest dose required for efficacy.

### 2.4. Evaluation and monitoring

Blood and spot urine samples (non-fasting) were collected during the morning hours (8AM-12:00PM) for biochemical analysis both at the initiation and end of each rifampin dose. Signs (dehydration, hypertension, failure to thrive, and muscular hypotonia) and symptoms (poor feeding, constipation, abdominal pain, polyuria, recurrent vomiting, lethargy, irritability, and seizure like symptoms) of hypercalcemia were assessed at each study visit [7], with careful monitoring for adverse events. At the initiation and completion of study, a complete physical examination was performed. Participants’ daily calcium and vitamin D, protein and calorie intake was assessed by the study’s registered dietician [21,22]. A renal ultrasound, at the initiation and completion of study, was completed and interpreted by experienced in-house pediatric radiologists. Presence of renal calcification, such as renal calculi and/or signs of nephrocalcinosis, as well as changes between the two measured time points were reported.

### 2.5. Assessment of rifampin compliance

Standard procedures were taken by research support pharmacy to assess the residual volume of rifampin suspension and pill counts post rifampin intake to determine adherence and compliance with study drug.

### 2.6. Biochemical analysis

Standard laboratory procedures were used in the Department of Pediatric Laboratory Medicine (DPLM) laboratory at The Hospital for Sick Children, Toronto, Canada to determine serum concentrations of albumin, calcium, ionized calcium, phosphate, alkaline phosphatase (ALP), AST, ALT, bilirubin, complete blood count, cortisol, ACTH, and creatinine as well as urinary calcium and creatinine levels. Total serum concentration of 25(OH)D was quantified by liquid chromatography-tandem mass spectrometry (LC-MS/MS) and intact PTH (iPTH) was assessed on the IDS-ISYS Multi-Discipline automated analyzer (Immunodiagnostic System, Boldon, UK) [23]. Total serum 1,25(OH)2D was measured on the Diasorin Liaison XL (Liaison, Saluggia, VC, Italy) at the Department of Clinical Laboratory at Mount Sinai Hospital, Toronto, Ontario. All results were compared with age- and sex-specific reference ranges [24].

Comprehensive analysis of vitamin D metabolites in serum was performed by Department of Pharmaceutics, University of Washington [17] using a chromatographic separation methodology that ensures complete separation of 1,25(OH)2D3 from 4β,25(OH)2D3, the principal product of CYP3A4 oxidation of 25(OH)D3 [16,25], as well as from 24 R,25(OH)2D3 and 23 S,25(OH)2D3, principal products of CYP24A1 oxidation of 25(OH)D3. Serum 4β-OH-cholesterol was measured by LC-MS/MS, as described [17].

### 2.7. Vitamin D metabolite ratios

Serum ratios including 1,25(OH)2D/PTH, 1,25(OH)2D/25(OH)D and 25(OH)D/ 24,25(OH)2D ratios were calculated at each study visit as proxy measures to estimate the PTH-dependent production of 1,25(OH) 2D, the conversion rate of 25(OH)D to 1,25(OH)2D, and CYP24A1-dependent clearance of 25(OH)D, respectively. [3, 26, 27]. Each metabolite/parent ratio is equal to the respective metabolite formation clearance/metabolite clearance. Ratios were compared with baseline results of a cohort of 82 healthy male and female controls age 18–60 years (ClinicalTrials.gov, NCT02019875). Serum 4β-OH-cholesterol levels as well as 4*β,*25(OH)2D/25(OH)D ratios were used as proxy measures of CYP3A4 activity as previously described [3,17].

### 2.8. Study outcomes

The primary outcome of the study was to evaluate the efficacy of rifampin in reducing serum concentrations of 1,25(OH)2D. The secondary outcomes included the reduction of serum calcium, urinary ca:cr and serum 1,25(OH)2D/PTH ratio as well as the change in 1α-hydroxylase and 24-hydroxylase activity and to compare the response between HCINF3 subjects and HCINF1 control.

### 2.9. Statistical analyses

In this exploratory pilot study, median percent changes from baseline were calculated for both types. Paired t-test was used to compare changes between baseline and post-treatment timepoints for the primary outcome. To compare results between HCINF1 and HCINF3 subjects, two-sided 95% confidence intervals were calculated and for the one patient with HCINF1 used as fix value. Nonparametric tests were used to assess potential statistical differences between HCINF1 and HCINF3 subjects.

## 3. Results

### 3.1. Study population

Six patients (P1-P6) aged six months to 17 years (male:2, female 4) consented to participate in the study. As seen in Table 1A, all participants underwent genetic testing as reported previously [3]. Participant 1 (P1) had biallelic pathogenic *CYP24A1* variants causing HCINF1 and was included as a control subject. Five participants (P2-P6) were classified as HCINF3 (in 3, one or more genetic variant was identified, whereas in 2, no genetic variant was found). Clinical features included renal calcification (3 participants), GI symptoms such as recurrent vomiting, constipation, and abdominal pain [2], poor feeding [3], irritability [1], and sclerosis of the metaphysis in the femur, tibia and fibula.

At baseline as seen in Table 1B, all participants had serum levels of calcium that were above the upper limit of the reference range for age, suppressed or low serum PTH levels, urinary ca:cr ratios above the upper limit, and elevated (or high normal) serum concentrations of 1,25(OH) 2D3.

### 3.2. Adherence to study design

The HCINF1 patient and four HCINF3 patients completed all the study visits as per protocol; one subject (P2) was discontinued from the study due to the development of an unrelated eating disorder (see below). None of the study participants missed a study visit, however, due to the COVID-19 pandemic situation, 2 study visits had to be rescheduled as virtual visits, with biological samples and biochemical analyses collected and performed in external laboratories (no vitamin D metabolites were measured). As shown in Table 2, compliance with the study drug was excellent in all study participants for both the 5 mg/kg/day and 10 mg/kg/day dosing with the lowest compliance observed in P5 (mean 92.8%). As well, most participants were able to increase their dietary calcium intake according to the study protocol to a DRI of 99.63 + /− 26.9%. All participants adhered to 200 IU of vitamin D supplements daily (DRI 64.9 ± .19%) while the vitamin D intake from diet remained variable (9.9 ± 33.84%).

### 3.3. Safety and Tolerability

All participants tolerated rifampin well as there was no clinical or biochemical evidence for hepatic dysfunction, adrenal insufficiency, or other symptoms that could be attributed to side effects of therapy. Two subjects reported one adverse event each: P3 had reduced appetite for 2 days during a viral illness, and P2 required hospitalization for a newly diagnosed eating disorder 6 weeks after stopping the low dose rifampin treatment. This participant was withdrawn from the study and was referred for assessment and treatment. Both adverse events were deemed as likely not to be related to the study intervention.

### 3.4. Biochemical outcomes

Biochemical responses including median percent change after treatment with 5 mg/kg/d as well as 10 mg/kg/d rifampin for primary and secondary outcomes are summarized in Table 3. In addition, biochemical response for each individual is provided in the Supplementary Table. Although the median percent change of 1,25(OH)2D from baseline to post-treatment timepoints were large overall, no statistical significance was reached due to the small sample size. Further, no significant differences were identified when comparing the HCINF1 and HCINF3 subjects possibly for the same reason.

#### 3.4.1. HCINF1(P1)

The control subject showed an increase in the 4β,25(OH)2D/25(OH) D ratio after rifampin 5 mg/kg/d (+60.56%) and 10 mg/kg/d (+78.3%) indicating a dose-dependent induction of CYP3A4 expression. Results were not available for the 4β-OH-cholesterol levels as not enough sample was available for quantification. Serum concentrations of 1,25(OH) 2D decreased on rifampin 5 mg/kg/d (−60.6%) and on 10 mg/kg/d (−32.8%). However, the serum calcium level was unchanged. As well, the urinary Ca:Cr ratio remained borderline high after both doses, respectively (+18.0%, +28.77%). The 1,25(OH)2D/PTH ratio decreased on both doses of rifampin (−44.8%, −32.8%) although ratios remained above the reference range. On the 5 mg/kg/day, serum concentrations of 25(OH)D decreased (−22.4%). Surprisingly, there was a paradoxical increase in 25(OH)D (+3.48%) on the higher dose of rifampin, 10 mg/kg/day, that was attributed to excessive sun exposure during the summer months. Both doses of rifampin led to decreases in calculated 1 alpha-hydroxylase activity (−49.3% vs −35.1%) and decreases in the calculated 24-hydoxylase activity.

(−57.5%vs −51.49%) although values remained very high compared with reference values.

#### 3.4.2. HCINF3 (P2-P6)

Percent changes observed at the end of the 5 mg/kg/d dosing period are provided for P2-P6. Percent changes observed at the end of the 10 mg/kg/d dosing period are provided for P3-P6 (P2 was withdrawn for this dosing period). CYP3A4 induction was observed at the end of both dosing periods with an increase in the ratio 4β, 25(OH)2D/25(OH) D (+96.83%, +56.68%), as well for the 4β-OH-Cholesterol (+169.27%, +55.93%). Nevertheless, the induction was less with the higher dose of rifampin than with the lower dose (see Discussion). There was a decrease in 1,25(OH)2D only with 10 mg/kg/day (−15.33%). On both doses, serum calcium remained high or borderline high (+0.76%,+0.54%) However, there was a decrease in urinary ca:cr ratio at the end of both dosing periods (−21.97%, −31.1%). At the end of both dosing periods, an increase in 1,25(OH)2D/PTH ratio was observed (15.03%, 11.81%). Serum concentrations of 25(OH)D3 did not change significantly after both doses, respectively (+7.07%, +19.45%) for the HCINF3 subjects. After both dosing periods, the calculated activity of 1alpha- hydroxylase decreased.

(−11.47%, −32.78%), and 24-hydroxylase activity increased (+25.97%, +14.79%).

### 3.5. Clinical outcomes

No symptoms of hypercalcemia or hypercalciuria were observed and no new renal calcification was identified. For P1, at the end of both dosing periods, there was an increase in weight percentile (+7.14%, +8.00%). For P2 through P6, at the end of the 5 mg/kg/d dosing period, there was a decrease in weight percentile (−29.88%), but at the end of the 10 mg/kg/d dosing period, there was an increase in weight percentile (+4.42%).

## 4. Discussion

The current treatment recommendations for biochemically active IIH patients includes some degree of vitamin D restriction (i.e., reducing dietary intake of vitamin D-rich or fortified foods, avoiding vitamin D contained supplements, and avoiding excess sun exposure) in combination with dietary calcium restriction. Although this approach is practical in formula-fed infants who can receive Calcilo-XD, these restrictions are far more difficult in older children and adults, suggesting a problematic long-term strategy, which may explain why renal calcification and insufficiency are common in this disorder [4,28,29]. Moreover, this dietary approach does not directly address the underlying problem of increased circulating concentrations of 1,25(OH)2D3 [7]. Vitamin D and calcium restriction may compound the problem in that both may increase 1α-hydroxylase activity resulting in persistently high or higher 1,25 (OH)2D3 levels. Impaired mineralization of intramembranous bone was found in a knockout mouse model of *Cyp24* deficiency [30]. It is therefore conceivable that chronically elevated concentrations of 1,25(OH)2D in carriers of biallelic pathogenic variants of *CYP24A1* may lead to depletion of calcium from bone thereby leading to osteopenia and maintenance of the hypercalcemia [3,31]. The consequence of these ongoing disturbances, especially the higher urinary calcium excretion, is a worsening of renal calcification with an associated risk of chronic renal impairment [18,32].

Pre-intervention, in the HCINF1 participant, 1-α-hydroxylase activity was at the lower end of the reference range, and 24 hydroxylase activity was markedly reduced, which are both findings consistent with the underlying mutation. In the HCINF3 participants, 1- α-hydroxylase activity, was in the mid-normal reference range. This implicates CYP27B1 in the pathophysiology of the increased 1,25(OH)2D3 levels in that the higher 1,25(OH)2D3 levels might be expected to downregulate CYP27B1. 24 hydroxylase activity was also in the mid-normal range. This latter finding suggests normal, but not increased (compensatory) CPY24A1 function.

Rifampin was used to induce CYP3A4 and thereby provided a supplementary pathway for clearance of vitamin D metabolites as previously described [17] Surprisingly, we found evidence for a greater effect of rifampin at the lower dose compared to the higher dose based on increases in circulation concentrations of 4β-OH-cholesterol. The basis for this observation is unclear but might be attributed to the sequential study design, the small number of subjects studied, and the intra-individual variability in hepatic CYP3A4 regulation, reduced adherence with rifampin during the second treatment period, or by other unknown factors.

Although rifampin reduced serum levels of 1,25(OH)2D3 in HCINF3 patients, the responses were quantitatively less than those of the control subject with HCINF1 who lacks the native pathway for inactivation of vitamin D metabolites provided by CYP24A1 [17,18]. These results suggest that patients with IIH who retain CYP24A1 activity do not benefit from induction of CYP3A4 as much as patients who lack CYP24A1, possibly related to higher production of 1,25(OH)2D3 in those with in HCINF3.

We previously proposed that an elevated 1,25(OH)2D/PTH ratio as a marker for PTH-independent action of 1,25(OH)2D, a finding that characterizes all forms of IIH [3]. Here we show that patients with HCNF3 have elevated 1,25(OH)2D/PTH ratio which does not decrease with rifampin and normal CYP24A1 activity, thereby suggesting that increased production of 1,25(OH)2D through a yet unknown mechanism. The 1,25(OH)2D/PTH ratio decreased only in the HCINF1 participants with both doses, although it remained markedly elevated in relation to the reference range in both types. The persistent inappropriate elevation of 1,25(OH)2D levels as indicated by the elevated 1,25(OH)2D/PTH ratio in both types likely accounts for the mild ongoing hypercalcemia and intermittent hypercalciuria. In addition, increased dietary calcium intake may have contributed as all patients had their dietary calcium intake liberalized for the study period.

A decrease in the 1,25(OH)2D/25(OH)D ratio was observed after rifampin use in both the HCINF1 and HCINF3 after both doses. This is best explained by an induction of 1,25(OH)2D clearance by rifampin, plausibly as a result of CYP3A induction in the liver and small intestine. CYP3A4 catalyzes 23R-, 24 S and 23S-hydroxylation of 1,25(OH)2D [33] and induction of these pathways of elimination by rifampin could produce the observed biochemical outcomes. The increase in 24-hydroxylase activity after rifampin use in Type 1 IIH is surprising, as in vitro studies suggests a repression of 24-hydroxylase activity with rifampin in individuals with normal *CYP24A1* gene [34]. A possible explanation for this finding is that the increased 24-hydroxylase activity is coming from a gene other than *CYP24A1* induced by rifampin (possibly *CYP3A4)* where this effect might only be measurable in individuals with biallelic *CYP24A1*-loss of function.

Overall, the biochemical response in both types of IIH was dose-dependent, with a quantitatively greater effect with rifampin 10 mg/kg/day compared to 5 mg/kg/day. Rifampin reduced the serum 1,25 (OH)D level and the 1,25(OH)2D/25(OH)D ratio at both doses in HCINF1, and at the 10 mg/kg dose for HCINF3. 1,25(OH)2D/PTH ratio was only reduced in HCINF1, whereas urinary ca:cr ratio only decreased in HCINF3 at both doses.

Among the strengths of the study is a well-characterized cohort with an established natural history of the condition. There was a strong commitment to the study design from study participants and their families. Since most of the participants were infants and toddlers, the confounding impact of the placebo effect was reduced. Due to the short treatment period, extraneous factors, such as intercurrent illness were minimized that might confound results.

This study has several limitations. Treatment duration was insufficient for the observation of a full treatment effect. The sample size was low therefore no randomization of subjects was possible. Further, the small sample size does not allow for a complete assessment of the reductions in 1,25(OH)2D level. The outpatient model in our study design did not allow for rigorous control of calcium and Vitamin D intake. The random urine calcium: creatinine ratio is not a valid index for 24- hour urine calcium excretion [35]. Lastly, serum levels of rifampin were not assessed preventing further understanding of rifampin pharmacodynamics.

In conclusion, the results of this pilot study suggest that rifampin use in HCINF1at a dose of 5 and 10 mg/kg for 2 months, reduces 1,25(OH) 2D levels, 1,25(OH)2D/PTH ratio and 1- α-hydrolase activity. These promising outcomes argue for further study to test the hypothesis that more prolonged use will result in greater efficacy. In HCINF3 participants, the reduction in 1,25(OH)2D and 1-α-hydroxylase activity with 10 mg/kg suggests that rifampin could be studied as adjuvant therapy in future studies.

## Supplementary Material

Supp Material

## Figures and Tables

**Table 1A:** Descriptive Characteristics including Genetic History and Clinical Presentation of Study Population.

	Proband 1 (P1)	Proband 2 (P2	Proband 3 (P3)	Proband 4 (P4)	Proband 5 (P5)	Proband 6 (P6)
**Age, sex**	14 y.o. male	5 y.o. female	23 m.o. male	20 m.o. female	3.5 y.o. female	44 m.o,female
**Weight, kg (Percentile)**	51.8 [42]	15.8[9]	12.4 [44]	9.1[7]	17.2 [74]	17.3 [79]
**Clinical Presentation**	Renal calcification	Recurrent vomiting, constipation, poor feeding	Poor feeding, irritability, renal calcification	Poor feeding,renal calcification	Constipation, abdominal pain	Sclerosis of the Metaphysis in the Femur, Tibia and Fibula
**Other Relevant Diagnosis**	-	-	Posterior urethral valves s/p ablation, bilateral Hydro-nephrosis	Congenital Diaphragmatic Hernia, Chronic Lung disease of Prematurity	Hypertension, Prematurity, Developmental Delay, Dysmorphism	Metaphyseal sclerosis
**Genetic Variant**	CYP24A1 (NM_000782.5): c.428–430del CYP24A1 (NM_000782.5): c.428–430del	None found	None found	SLC12A1 (NM_000338.2): c .262 G>A (p.Ala88Thr) CASR(NM_001178065.1): c .1882 C>T (p. Leu628Phe)	SLC4A1 (NM_000342.2): c .1151 G>A (p.ARg384His)	SLC4A1 c1480G>A (p.Gly494Ser) SLC26A1 c .1981 G>A (p.Gly661Ser)

**Table 1B:** Baseline Biochemical Characteristics of Study Population.

	Proband 1 (P1)	Proband 2 (P2)	Proband 3 (P3)	Proband 4 (P4)	Proband 5 (P5)	Proband 6 (P6)	Reference Range
**Biochemistry & Vitamin D Metabolites** **Total calcium (mmol/L)**	2.88	2.97	2.74	2.85	2.67	2.58	2.22–2.54
**iPTH (pmol/L)**	*<* 0.53	3.18	3.39	1.70	0.95	1.06	1.27–9.33
**Creatinine (umol/L)**	70.00	27.00	29.00	14.00	19.00	28.00	8.00–89.00
**Urinary ca:cr ratio (mmol/mmol)**	1.35	0.58	0.33	0.73	2.40	0.70	**
**25(OH)D (nmol/L)** ^a^	160.99	86.11	70.64	62.90	66.52	44.58	14.50–121.00
**25(OH)D (ng/L)** ^a^	64.50	34.50	28.30	25.20	26.65	17.86	36.25–302.00
**24,25(OH)2D (ng/L)**	0.14	3.33	4.68	2.50	2.36	1.72	0.75–33.3
**1,25 (OH)2D (pmol/L)**	187.00	261.00	239.00	167.00	154.00	191.00	48.00–190.00
**4β,25 (OH)2D (pmol/L)**	528.03	434.43	219.61	146.65	292.63	78.25	19.40–481.00
**4β-OH-Cholesterol (nmol/L)**	-	126.91	179.56	117.97	201.66	166.89	406.00 ± 85.00
**Vitamin D Metabolite Ratios** **1,25 (OH)2D/PTH (pmol/pmol)**	352.83^b^	82.08	70.46	98.47	161.43	180.19	3.60 − 39.80
**1,25 (OH)2D/ 25(OH)D (pmol/nmol)** ^c^	1.16	3.03	3.38	2.66	2.32	4.28	0.73–5.10
**25(OH)D/24,25 (OH)2D (ng/ng)** ^d^	460.71	10.36	6.05	10.08	11.27	10.38	5.11–51.00
**4β,25 (OH)2D/25(OH)D (pmol/nmol)** ^c^	3.28	5.04	3.11	2.33	4.40	1.76	0.92–8.21

* * age dependent *<* 2.5 (0–6mo), *<* 1.8 (6–12 mo), *<* 0.6 (>12 mo)

a The value for 25(OH)D is reported in units of nmol/L (obtained at the Hospital for Sick Children) and ng/L (obtained at Department of Pharmaceutics, University of Washington)

b Ratio was calculated using the lower limit concentration of the assay.

c Ratio was calculated using the 25(OH)D level from Hospital for Sick Children.

d Ratio was calculated using the 25(OH)D levels obtained at Department of Pharmaceuticsm, University of Washington

**Table 2:** Adherence of Study Population to Study Design.

	Baseline 1 prior to 5 mg/kg/d (Median ± SD)	End of 5 mg/kg/ d Treatment (Median ± SD; [Median % Change])	Baseline 2 prior to 10 mg/kg/d (Median ± SD)	End of 10 mg/kg/d Treatment (Median ± SD; [Median % Change])
**Study Population**	**n = 6 (P1&P2-P6)**	**n = 6 (P1&P2-P6)**	**n = 5 (P1&P3-P6)**	**n = 5 (P1&P3-P6)**
Calcium Intake (% DRI) *(P1) (P2-P6)*	29 65 ± 19.53	41.5[+ 43.1] 100 ± 41.04 [+ 53.85]	62 80 ± 19.38	149.3 [+ 140.80] 98.82 ± 20.42 [+ 23.5]
Vitamin D Intake from Diet (% DRI) *(P1) (P2-P6)*	2.5 27.03 ± 27.60	0 [−100.0] 26.8 ± 4.14	26.7 22.55 ± 14.57	95 [+ 255.81] 9.05 ± 5.91 [−59.87]
Vitamin D Intake with Supplementation (% DRI) *(P1) (P2-P6)*	Not assessed	33 [N/A] 60.25 ± 4.20 [N/A]	0 44 ± 13.17	128.3 [+ 1283] 53.2 ± 26.17 [+ 20.9]
Compliance Assessment (%) *(P1) (P2-P6)*	Not assessed	94.95^a^ 106.28 ± 12.40	Not assessed	96.3^a^93.99 ± 5.65

a For P1, compliancy is reported as a mean

**Table 3:** Biochemical responses of study population with 5 mg/kg/d as well as 10 mg/kg/d rifampin for primary and secondary outcomes.

	Baseline 1 prior to 5 mg/kg/d (Median ± SD)	End of 5 mg/kg/d (Median ± SD; [% Change])	Baseline 2 prior to 10 mgkg/d (Median ± SD)	End of 10 mg/kg/d (Median ± SD); [% Change])	Reference Range
**Study Population**	**n = 6** **(P1&P2-P6)**	**n = 6** **(P1&P2-P6)**	**n = 5** **(P1& P3-P6)**	**n = 5** **(P1& P3-P6)**	
**Biochemistry & Vitamin D Metabolites**					
Total Calcium (mmol/L) (P1) (P2-P6)	2.60 2.63 ± 0.15	2.62 (+0.8) 2.65 ± 0.13 [+ 0.76]	2.50 2.65 ± 0.11	2.57 (+2.8) 2.65 ± 0.09 [+ 0.54]	2.22–2.54
iPTH (pmol/L) (P1) (P2-P6)	0.74 2.12 ± 0.81	0.53 (−28.40) 2.00 ± 1.12 [−5.66]	0.53 2.07 ± 0.72	0.53 (0.00) 2.49 ± 1.46 [+ 20.29]	1.70–9.33
Urine Ca:Cr (mmol/mmol) (P1) (P2-P6)	0.39 0.66 ± 0.35	0.46 (+18.0) 0.52 ± 0.35 [−21.97]	0.73 1.03 ± 1.01	0.94 (+28.77) 0.71 ± 0.77 [−31.1]	a
25(OH)D (nmol/L)^a^ (P1) (P2-P6)	125.00 66.52 ± 13.38	97.00 (−22.4) 71.23 ± 13.45 [+ 7.07]	86.00 55.01 ± 5.89	89.00 (+3.48) 65.71 ± 13.01 [+ 19.45]	14.50–121.00
25(OH)D (ng/L)^a^ (P1) (P2-P6)	64.5 26.65 ± 5.36	29.4 (−54.42) 28.54 ± 5.39 [+ 7.09]	27.7 22.04 ± 2.36	43.3(+56.32) 30.32 ± 4.09 [+ 37.57]	36.25–302.00
24,25(OH)2D (ng/L) (P1) (P2-P6)	0.14 5.66 ± 0.55	0.15(7.14) 5.26 ± 7.98 [−7.07]	0.09 4.75 ± 0.39	0.29 (+222) 4.34 ± 0.54 [−8.63]	0.75–33.30
1,25(OH)2D (pmol/L) (P1) (P2-P6)	350 154 ± 40.83	138 (−60.6) 174.50 ± 41.09 [+ 13.31]	262.00 150.00 ± 33.41	176.00 (−32.8) 127.00 ± 26.62 [−15.33]	48.00–190.00
4β,25(OH)2D (pmol/L) (P1) (P2-P6)	528.03 219.61 ± 123.05	386.42(−26.82) 388.82 ± 134.51 (+77.1)	202.33 173.89 ± 31.06	564.04 (+178.77) 290.54 ± 25.42 [+ 67.09]	19.40–481.00
4β-OH-Cholesterol (nmol) (P1) (P2-P6)	- 166.89 ± 31.69	- 449.40 ± 148.17 [169.27]	- 197.19 ± 40.33	406.1 (N/A) 307.47 ± 45.72 [+ 55.93]	406.00 ± 85.00
**Vitamin D Metabolite Ratios**					
1,25(OH)2D/PTH (pmol/pmol) (P1) (P2-P6)	471.69 87.00 ± 45.13	260.38 (−44.8) 100.08 ± 40.91 [+ 15.03]	494.34 72.82 ± 33.77	332.08 (−32.8) 81.43 ± 23.70[+ 11.82]	3.6–39.80
1,25(OH)2D/25(OH)D (pmol/nmol)^b^ (P1) (P2-P6)	2.80 2.66 ± 0.53	1.42 (−49.3) 2.36 ± 0.52 [−11.47]	3.05 2.41 ± 0.43	1.98 (−35.1) 1.62 ± 0.44 [−32.78]	0.73–5.10
25(OH)D/24,25(OH)2D(ng/ng)^c^ (P1) (P2-P6)	460.71 10.36 ± 1.84	196(−57.46) 13.05 ± 1.95 [+ 25.97]	307.78 10.75 ± 2.54	149.31(−51.49) 12.34 ± 3.50 [+ 14.79]	5.11–51.00
4β,25(OH)2D/25(OH)D (pmol/nmol)^b^ (P1) (P2-P6)	3.28 3.11 ± 1.23	5.27 (+60.67) 6.12 ± 1.06 [+ 96.78]	2.93 2.85 ± 0.48	5.22 (+78.16) 4.17 ± 2.12 (+46.32)	0.92–8.21

a The value for 25(OH)D is reported in units of nmol/L (obtained at the Hospital for Sick Children) and ng/L( obtained at Department of Pharmaceutics, University of Washington)

b Ratio was calculated using the 25(OH)D level from Hospital for Sick Children

c Ratio was calculated using the 25(OH)D levels obtained at Department of Pharmaceuticsm, University of Washington

a age dependent *<* 2.5 (0–6mo), *<* 1.8 (6–12 mo), *<* 0.6 (>12 mo)

## Data Availability

Data will be made available on request.

## Abbreviations:

IIH: Idiopathic Infantile Hypercalcemia
HCINF1: infantile hypercalcemia-1
VUS: variance of uncertain significance
Ca:cr: calcium to creatinine
DRI: daily recommended intake
ALP: alkaline phosphatase
